# Antiphospholipid Antibody Syndrome-Associated Increased Surface Expression of VLA4 Integrin on Human Monocytes

**DOI:** 10.3390/biomedicines10102341

**Published:** 2022-09-20

**Authors:** Ula Štok, Neža Štucin, Elizabeta Blokar, Aleš Ambrožič, Snežna Sodin-Šemrl, Saša Čučnik, Polona Žigon

**Affiliations:** 1 Department of Rheumatology, University Medical Centre Ljubljana, SI-1000 Ljubljana, Slovenia; 2 Faculty of Pharmacy, University of Ljubljana, SI-1000 Ljubljana, Slovenia; 3 Faculty of Medicine, University of Ljubljana, SI-1000 Ljubljana, Slovenia; 4 Faculty of Mathematics, Natural Sciences and Information Technologies, University of Primorska, SI-6000 Koper, Slovenia

**Keywords:** antiphospholipid syndrome, catastrophic antiphospholipid syndrome, monocytes, adhesion molecules, selectins, integrins, VLA4

## Abstract

Antiphospholipid syndrome (APS) is a systemic autoimmune disorder characterized by thrombosis and/or obstetric complications in the presence of antiphospholipid antibodies (aPL). Catastrophic APS (CAPS) is the most severe form of the disease, in which microvascular thromboses develop rapidly, leading to multiorgan failure. Monocytes, along with endothelial cells, are critical players in the pathogenesis of APS. Recruitment of these cells to the site of injury/inflammation involves a series of events, including capture, rolling, adhesion enhancement, and transmigration, which are controlled by surface adhesion molecules. The aim of our study was to investigate the surface adhesion profile of monocytes from APS patients and monocytes stimulated in vitro with aPL from a CAPS patient. The surface expression of the adhesion molecules LFA1, L-selectin, MAC1, PSGL1, and VLA4 was analyzed by flow cytometry. To our knowledge, this preliminary study was the first to show that VLA4 was significantly increased on the surface of monocytes from APS patients. Moreover, in vitro stimulations mimicking CAPS showed an even greater increase in VLA4. Our data suggest that the surface adhesion profile on monocytes is altered in APS and CAPS and may be involved in the thrombotic pathophysiology of the disease by enhancing monocyte adhesion.

## 1. Introduction

Antiphospholipid syndrome (APS) is a systemic autoimmune disorder characterized by thrombosis and/or obstetric complications in the continuous presence of antiphospholipid antibodies (aPL) [1]. aPL are a heterogeneous group of autoantibodies, of which anti-cardiolipin (aCL), anti-β2 glycoprotein I (a-β2GPI), and lupus anticoagulant (LA) are currently among the laboratory classification criteria for APS [2]. In addition, other non-criteria aPL, such as antibodies against phosphatidylserine/prothrombin complex (aPS/PT), have been found to play an important role in APS [3,4]. Catastrophic APS (CAPS) is extremely rare, but the most severe form of the disease, in which accelerated and widespread thrombosis, leads to multipleorgan failure [5].

The pathogenesis of APS and CAPS is not fully understood, but apparently involves a complex interaction of aPL with endothelial cells, platelets, and monocytes [6]. Interaction of aPL with monocytes triggers their pro-inflammatory and pro-coagulant phenotype, resulting in increased expression of pro-inflammatory molecules such as TNF-α and IL1-β, as well as in increased expression of tissue factor (TF) both in vitro [7,8,9] and in vivo [10,11,12]. Moreover, exposure of endothelial cells to aPL upregulates the expression of monocyte chemoattractant protein 1 (MCP-1), which stimulates the synthesis of TF in monocytes [13]. aPLs promote adhesion between leukocytes and endothelial cells and thrombosis in vitro [14,15] and in vivo in mice [16].

Monocytes contribute fundamentally to immune surveillance and the inflammatory response both in normal physiological states and in disease. Transmigration of monocytes to sites of injury/inflammation is a tightly regulated, multistep process involving a series of interactions between immune cells and the endothelium and requiring various adhesion molecules. It involves a series of events, including capture, rolling, adhesion enhancement, and lateral locomotion of monocytes [17]. In the first step, selectins (P- and E-selectin) on the endothelium interact with their dominant ligand P-selectin glycoprotein ligand-1 (PSGL1) on monocytes and L-selectin (CD62L) on monocytes interacts with carbohydrates presented by specific ligands of endothelial cells. These interactions enable monocyte tethering and rolling along the vascular endothelium [18]. Importantly, the binding mediated by selectins is weak. Therefore, stronger integrin-mediated binding is required for monocytes to withstand the high shear stress imposed by blood flow and attach firmly to endothelium and migrate further into the tissue. The best studied integrins that contribute importantly to the monocyte adhesion cascade include very late antigen 4 (VLA4; CD49d/CD29; integrin α4β1), macrophage receptor 1 (MAC1; CD11b/CD18; integrin αMβ2), and lymphocyte function-associated antigen 1 (LFA1; CD11a/CD18; αLβ2 integrin) [17].

Classification of human monocytes into three subsets has been proposed according to the relative abundance of two surface markers: CD14++CD16- or “classical” (80–95%), CD14++CD16+ or “intermediate” (2–11%), and CD14+CD16++ or “non-classical” (2–8%). These subsets are functionally and phenotypically distinct [19]. Despite advances in phenotypic analysis, immune functions associated with the specific monocyte subset remain poorly defined and reports in the literature are inconsistent [20]. Intermediate monocytes have been described to release pro-inflammatory cytokines upon stimulation [21,22], but increased secretion of anti-inflammatory cytokines has also been found in the intermediate subset [23] and in the classical subset [24,25]. On the other hand, the non-classical subset was described as pro-inflammatory [23]. Therefore, it is difficult to assign the pro- or anti-inflammatory state of monocytes upon activation to specific subsets.

While data on the surface adhesion profile of monocytes in APS are limited, there is some evidence of deregulated genes and altered miRNA profiles involved in triggering cell adhesion and migration to sites of inflammation in APS monocytes [26]. Therefore, the main objective of our study was to determine the surface expression of the five best studied adhesion molecules of the monocyte adhesion cascade LFA1, L-selectin, MAC1, PSGL1, and VLA4 on monocytes from APS patients. In addition, we aimed to investigate the key molecules differentially expressed on whole blood monocytes in vitro by mimicking the most severe form of APS—CAPS.

## 2. Materials and Methods

### 2.1. Literature Search and Mining

The literature search was performed in the MEDLINE bibliographic database using PubMed^®^ and the following search terms: “antiphospholipid syndrome” AND “adhesion molecules”. Bibliometric analysis was performed using VOSviewer software (version 1.6.18) based on the frequent occurrence of authors’ keywords in MEDLINE search.

### 2.2. Study Design and Participants

**Figure 1:**
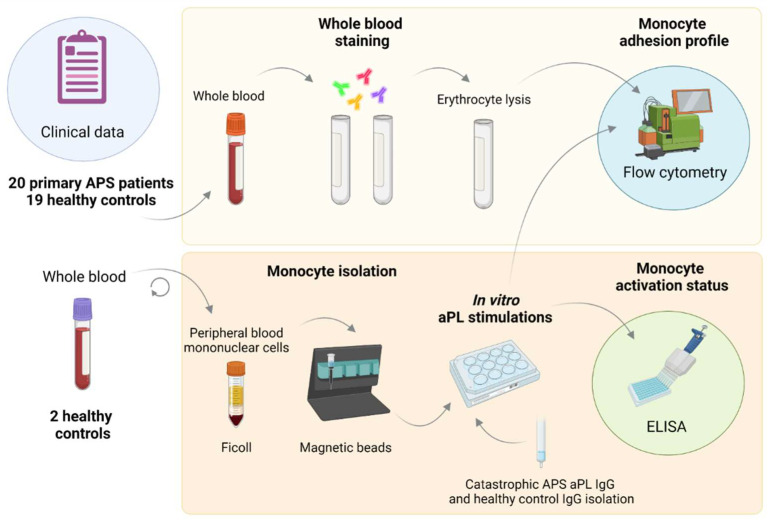
**Schematic representation of the study protocol.** Clinical and demographic data were collected. Whole blood was drawn and used to perform whole blood fluorescent staining (top panel) and in vitro CAPS aPL stimulations of isolated monocytes (bottom panel). Surface expression of adhesion molecules on both monocytes from APS patients and in vitro stimulated monocytes was determined by flow cytometry. In vitro monocyte activation was measured by ELISA. This figure was created using Biorender.com (accessed on 24 August 2022).

A total of 20 patients were recruited from the Department of Rheumatology, University Medical Centre Ljubljana. All patients met the updated international classification criteria for APS [1]. The study also included 19 age- and sex-matched healthy controls (HC). The study protocol is summarized in Figure 1 (top panel). Participants’ demographic (age, sex, and body mass index) and clinical data (history of venous, arterial, or microvascular thromboses; obstetric complications; presence of hyperlipidemia, hypertension, and diabetes) were systematically recorded. Treatment status was recorded (anticoagulation, antiaggregation, antimalarial medication).

For in vitro studies, pooled monocytes were isolated from two healthy volunteers and stimulated with IgG fractions isolated from a patient with CAPS suffering from venous and arterial thrombosis or a HC (Figure 1 bottom panel).

Informed consent was obtained from all subjects involved in the study. The study was conducted according to the guidelines of the Declaration of Helsinki and approved by the Ethics Committee of the Republic of Slovenia (0120-7/2019/5).

### 2.3. Antiphospholipid Antibodies Determination

Whole blood was collected in plain blood collection tubes and centrifuged at 1.800× *g* for 10 min at room temperature to obtain serum for determination of participants’ aPL profile, including LA, aCL, anti-β2GPI, aPS/PT of IgG, IgM, and IgA isotypes. All measurements were performed according to previously described protocols; aCL [27], anti-β2GPI [28,29], and aPS/PT ELISA [30]. A value above the 99th percentile of the healthy control population was considered significant. For measurement of LA, whole blood was collected in tubes containing citrate as an anticoagulant and centrifuged at 2000× *g* for 15 min at 15 °C to obtain platelet-poor plasma. Samples were filtered and stored at −80 °C until further use. Clotting tests were performed with a coagulation analyzer CS-2500 (Sysmex, Kobe, Japan) and STart (Diagnostica Stago, Asnières sur Seine Cedex, France), according to the guidelines of the International Society on Thrombosis and Haemostasis ISTH. We performed the Dilute Russell’s Viper Venom Test (dRVVT) test with the LA1 screening reagent and LA2 confirmatory reagent (Siemens Healthlineers, Erlangen, Germany) following the manufacturers’ instructions. Staclot LA (Diagnostica Stago, Asnières sur Seine Cedex, France) was used for LA detection/confirmation.

### 2.4. Purification of Serum aPL

Total IgG purification was performed from serum of a CAPS patient and a HC using HiTrap Protein G HP antibody purification columns (Cytiva, MA, USA) according to the manufacturer’s protocol. Flow-through samples were adjusted pH to a final pH of 6–7 with Tris–HCl buffer. Protein concentration measurements (A280 nm) on these fractions were used to establish the final pooled sample. After purification, IgG was dialyzed and concentrated to 1 mg/mL on an Amicon^®^ concentrator with a molecular mass cut-off of 100 kDa and stored at −80 °C. IgG fractions were tested for the aPL positivity using our in-house aCL [27], anti-β2GPI [28,29], and aPS/PT ELISA [30].

### 2.5. Whole Blood Staining

Venous blood from 20 APS patients and 19 HC was collected to vacutainer tubes containing heparin and processed within one hour. Whole blood (100 µL) was stained with a cocktail of fluorochrome-conjugated antibodies for 15 min at 4 °C in the dark. Monocyte subsets were identified by their size and granularity (FSC/SSC) and surface expression of CD14 and CD16 using fluorochrome labelled antibodies; anti-CD14-FITC (1:50, clone REA599, Miltenyi Biotech, Bergisch Gladbach, Germany) and anti-CD16-PE (1:40, clone eBi-oCB16, eBioscience, Thermo Fisher Scientific, MA, USA). Adhesion profiles were assessed with fluorochrome-labelled antibodies divided into two panels: first panel anti-CD11a-PEVio770 (1:44, clone REA378), anti-CD11b-APC (1:40, clone ICRF44), and CD49d-VioBlue (1:50, clone MZ18-24A9), and the second panel anti-CD62L-PEVio770 (1:200, clone 145/15) and anti-CD162-APC (1:22, clone REA319) (all from Miltenyi Biotech, Bergisch Gladbach, Germany). After washing with 2 mL of 0.5% BSA-FACS buffer, samples were lysed and fixed with whole blood lysis reagent (Beckman Coulter, CA, USA), according to the manufacturer’s protocol. After washing and resuspending cells in 0.5% BSA-FACS buffer, samples were measured on MACSQuant^®^ Analyzer 10 (Miltenyi Biotech, Bergisch Gladbach, Germany) and analyzed with FlowJo V10 software (Becton Dickinson, NJ, USA). Fluorescence minus one (FMO) was used to set up a negative gate.

### 2.6. Cell Experiments

#### 2.6.1. Purification of Human Monocytes from Healthy Controls

Peripheral blood mononuclear cells (PBMCs) were isolated from two healthy volunteers by gradient centrifugation using Ficoll-Paque Plus (Cytiva, MA, USA). Briefly, 30 mL of whole blood was collected in vacutainer tubes containing EDTA and immediately inverted. The whole blood was then diluted in PBS (1:2), loaded onto the Ficoll gradient, and centrifuged at 400× *g* for 25 min at room temperature using no brake. The buffy coat layer was carefully collected into 15 mL conical tubes and washed twice with PBS by centrifugation at 300× *g* for 10 min. Cell viability and number were determined by staining with trypan blue using an automated cell counter (Countess, Thermo Fisher Scientific, MA, USA). Monocytes were purified by negative selection using the Pan Monocyte Isolation Kit (Miltenyi Biotech, Bergisch Gladbach, Germany) according to the manufacturer’s protocol. The isolated monocytes were cultured in RPMI-1640 (STEMCELL Technologies, Vancouver, BC, Canada) supplemented with 2 mM L-glutamine (Lonza, Basel, Switzerland) in humidified atmosphere (5 vol. % CO_2_, 37 °C) for 2 h.

#### 2.6.2. In Vitro Stimulation of Monocytes from Healthy Controls with IgG Fractions

Cells were seeded in a Nunc UpCell 12 Multidish (Thermo Fisher Scientific, MA, USA) at a concentration of 0.5 × 10^6^ cells per well. To mimic the “two-hit” hypothesis, cells were stimulated with 25 ng/mL lipopolysaccharide (LPS from E. Coli O111:B4, Sigma Aldrich, MO, USA), 100 µg/mL IgG fractions isolated from serum of a patient with CAPS (aCL-, anti-β2GPI-, and aPS/PT-positive) or from sera of a HC, and 45 µg/mL of human prothrombin (Enzyme Research Laboratories, IN, USA). Final concentrations were determined after titration of the reagents.

#### 2.6.3. Flow Cytometry

Monocytes in 12-well plates were transferred to room temperature for 30 min to allow the cells to detach. Cells were collected and centrifuged at 300× *g* for 2 min at room temperature. The supernatant was collected and stored at −40 °C. The pelleted cells were resuspended in 200 µL of 0.5% BSA-FACS buffer, transferred to a V-bottom 96-well plate (Corning, NY, USA), and centrifuged at 300× *g* for 1 min at +4 °C. Cells were resuspended in 50 µL FACS buffer and stained with anti-CD14-FITC (1:50, clone REA599), anti-CD16-PE (1:40, clone eBi-oCB16), and CD49d-VioBlue (1:50, clone MZ18-24A9), all from Miltenyi Biotech (Bergisch Gladbach, Germany), for 15 min at 4 °C in the dark. After washing and resuspending cells in FACS buffer, samples were measured on MACSQuant^®^ Analyzer 10 (Miltenyi Biotech, Bergisch Gladbach, Germany) using FlowJo V10 software (Becton Dickinson, NU, USA) for analysis. FMO was used to set up a negative gate.

#### 2.6.4. Soluble TNF-α ELISA and IL-6 ELISA

The concentrations of soluble TNF-α and IL-6 in the cell culture media were determined using ELISA MAX™ Deluxe Set Human TNF-α and ELISA MAX™ Deluxe Set Human IL-6 (both BioLegend, CA, USA), respectively, according to the manufacturer’s protocol.

### 2.7. Statistical Analysis

An independent sample Mann–Whitney test was used to compare differences in the surface expression of adhesion molecules on monocytes from APS patients and HC. For in vitro studies a Student’s t-test was performed to compare differences in mean TNF-α, IL-6 and VLA4 levels after stimulating monocytes with CAPS or HC IgG. Statistical analyses were performed using IBM SPPS Statistics software, version 20 (IBM, Armonk, NY, USA), and all graphs were created using GraphPad Prism software, version V.9 (Dotmatics, MA, USA).

## 3. Results

### 3.1. Bibliographic Analysis

**Figure 2:**
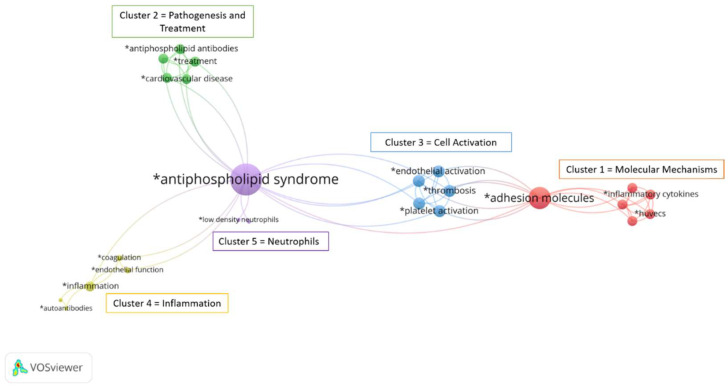
**A bibliometric analysis of the published literature on adhesion molecules and APS using VOSviewer software**. Mapping of keywords of studies pertaining to “adhesion molecules” and “APS” categorized into five clusters (molecular mechanisms, pathogenesis and treatment, cell activation, inflammation, and neutrophils).

To identify and visualize the foci in the published literature on adhesion molecules and APS, we performed a bibliographic analysis. The literature search in the MEDLINE database using PubMed^®^ and the keywords “APS” and “adhesion molecules” yielded 116 publications from 1995 to 2022. The authors’ keywords in the 116 publications were analyzed using the VOSviewer software (Leiden University, Leiden, The Netherlands) (Figure 2). A total of 110 keywords (e.g., related to processes, molecules, cell types, disease, etc.) that appeared at least once in the abstract or title were considered. The higher the number of coincidences, the closer the two terms were in the scheme. Of the total 110 keywords, 60 were excluded because they were irrelevant to the topic of our search, and 50 keywords were selected for analysis. Of the 50 selected keywords, the largest set of related terms consisted of 24 keywords organized into 5 clusters (molecular mechanisms, pathogenesis and treatment, cell activation, inflammation, and neutrophils). We showed that the published literature on adhesion molecules in APS focuses on different cell types involved in the pathogenesis of APS, namely platelets, endothelial cells, and neutrophils. Importantly, we have shown that previous publications and data on monocytes and their adhesion profile in APS are very limited because monocytes are not present in any of the clusters, especially since inflammation is fairly strongly represented in this scheme.

### 3.2. Demographic and Clinical Characteristics of APS Patients and Healthy Controls

Clinical and demographic characteristics of participants are shown in Table 1. APS patients and HC were sex, age, and BMI matched. All patients had primary APS of whom 6 (30%) had a history of arterial thrombosis, 11 (55%) of venous thrombosis, and 3 (15%) obstetric complications. Nine patients (45%) had hyperlipidemia, 5 (25%) hypertension, and none were diabetic. Sixteen patients (80%) received anticoagulation therapy, 2 (10%) received antiaggregation therapy, and 4 (20%) received antimalarial medication. Fourteen patients (70%) were positive for aCL antibodies, 13 (65%) had anti-β2GPI antibodies, and 10 (50%) had antibodies against PS/PT complex. Ten (50%) patients tested positive for all three types of antibodies, six (30%) were double positive, and four (20%) were single positive. Seventeen (85%) were positive for lupus anticoagulants (LA). Only HC that did not report thromboses, obstetric complications, hyperlipidemia, hypertension, or diabetes, had no other diagnosed rheumatic disorder, were not taking any medications at that time, and tested negative for aPL were included in the study.

### 3.3. Identification of Monocyte Subsets in Whole Blood

**Figure 3:**
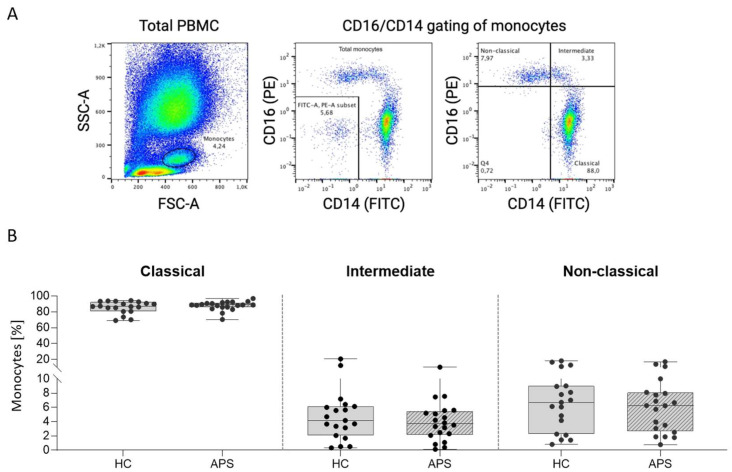
**Identification of blood monocyte subsets by flow cytometry.** (**A**) Gating strategy for identification of monocyte subsets in whole blood based on conventional bivariate scatterplots of CD16 vs. CD14. (**B**) Distribution of monocyte subsets between APS patients and HC. APS, antiphospholipid syndrome; FITC, fluorescein isothiocyanate; FSC, forward scatter; HC, healthy control; PBMC, peripheral blood mononuclear cells; PE, R-phycoerythrin; SSC, side scatter.

To determine the adhesion profile of monocytes, we analysed whole blood from APS patients and HC by flow cytometry. Monocytes were gated on a bivariate plot of forward scatter (FSC) vs. side scatter (SSC) signal (Figure 3A). To further identify the population of total monocytes, we used Boolean NOT gate on bivariate plot of CD14 vs. CD16 negative signal (Figure 3A). This population was further discriminated according to CD14 vs. CD16 surface expression to obtain three monocyte subsets. Approximately 90% of the total monocytes comprised the classical CD14++/CD16- subtype, about 4% intermediate CD14++/CD16+ subtype, and 6% comprised the non-classical CD14+/CD16++ subtype. The percentages of specific monocyte subsets were not significantly different between APS patients and HC (Figure 3B).

### 3.4. Differential Expression of Surface Adhesion Molecules on Total Monocytes and Monocyte Subsets

Both total monocytes and each specific subset were analysed for a positive signal for different integrins (LFA1, MAC1, and VLA4), L-selectin, and a PSGL1 (Table 2). We demonstrated that all adhesion molecules were present on the surface of monocytes and monocyte subsets. Surface expression of VLA4 was significantly increased (median percent positive (IQR)) on classical monocyte subset in APS patients 38.6 (31.2) compared with HC 23.0 (14); *p* = 0.038 and on intermediate subset in APS 84.1 (28.4) compared with HC 64.2 (30.4); *p* = 0.038 (Table 2). Similarly, the median fluorescence intensity (MFI) of VLA4 was also significantly increased on classical and intermediate monocytes in APS patients compared with the HC. The surface expression of LFA1, L-selectin, MAC1, and PSGL1 did not differ significantly between APS patients and HC (Table 2).

### 3.5. Mimicking Catastrophic APS In Vitro Significantly Increases Surface VLA4

**Figure 4:**
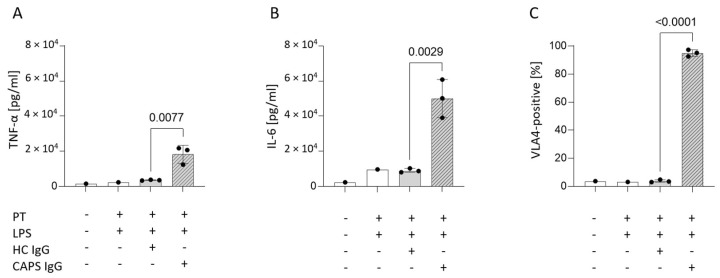
**Mimicking catastrophic APS in vitro.** Levels of soluble TNF-α (**A**) and IL-6 (**B**) in cell culture supernatants and VLA4 (**C**) surface expression after stimulation with CAPS IgG or HC IgG for 16 h. Untreated cells were completely naive while PT was added to all other stimulations since the CAPS IgG also contained high levels of aPS/PT antibodies. LPS and PT alone had minimal effect therefore combination of both was used as a background. The bars represent mean ± SD in three technical replicates. Statistical differences between CAPS and HC IgG stimulations were assessed by student’s t-test (*p* < 0.05). APS, antiphospholipid syndrome; CAPS, catastrophic APS; HC, healthy control; IgG, immunoglobulin G; IL-6, interleukin 6; LPS, lipopolysaccharide; PT, prothrombin; TNFα, tumour necrosis factor alpha; VLA4, very late antigen-4.

We stimulated isolated human monocytes pooled from two healthy volunteers with either aPL, an IgG fraction isolated from the serum of a patient suffering from CAPS, or an IgG fraction isolated from a HC serum in the presence of LPS and prothrombin. The patient with CAPS tested positive for aCL IgG (>30 AU), anti-β2GPI IgG (>16 AU), and aPS/PT IgG (100 AU), whereas HC tested negative for all three aPL. aPL significantly increased monocyte activation as determined by soluble TNF-α (Figure 4A) and IL-6 (Figure 4B) in cell culture supernatants. In addition, the percentage of VLA4-positive cells (± SD) increased significantly when monocytes were stimulated with CAPS IgG (95.07 ± 2.40) compared with HC IgG (3.79 ± 0.9); *p* < 0.0001 (Figure 4C).

## 4. Discussion

A bibliometric analysis of the published literature on adhesion molecules and APS revealed that research to date has focused on five distinct topics: Molecular Mechanisms, Pathogenesis and Treatment, Cell Activation, Inflammation, and Neutrophils. Surprisingly, monocytes did not appear in any of these topics, suggesting that data on monocyte adhesion molecules in APS are very limited. The only study on this topic that we found reported deregulated genes and altered miRNA profile involved in triggering cell adhesion and migration to sites of inflammation in APS monocytes [26]. Therefore, we set our study out to investigate the potentially altered surface expression of the five most studied adhesion molecules of the monocyte adhesion cascade, including LFA1, L-selectin, MAC1, PSGL1, and VLA4 on monocytes from APS patients. In addition, we investigated key molecules differentially expressed on APS whole blood monocytes in vitro by mimicking the most severe form of APS−CAPS.

We demonstrated that surface expression of VLA4 was significantly increased on monocytes from APS patients compared with monocytes from HC, suggesting that VLA4 plays an important role in monocyte adhesion even in the absence of an acute event. On the other hand, the surface expression of VLA4 on monocytes in vitro was even higher when CAPS was mimicked, suggesting that VLA4 also plays an important role at CAPS. VLA4 is a heterodimer of CD49d and CD29 and binds to its counter ligand VCAM-1, allowing monocytes to arrest on inflamed endothelium. To our knowledge, surface expression of VLA4 on monocytes from APS patients has not been studied. However, significantly increased levels of soluble VCAM-1 have been found in the plasma of APS patients compared with HC [31,32], which may be related to the higher surface expression of VLA4 that we see on monocytes from APS patients. In our study, the percentage of VLA4-positive cells in APS patients was highest in the intermediate monocyte subset, which has been reported to have a pro-inflammatory function [21,22]. Previous in vitro studies have shown that aPL from APS patients elicit a pro-inflammatory profile of monocytes [7,8,9]. If the intermediate monocyte subset is indeed pro-inflammatory, it is not surprising that surface expression of VLA4 was highest in this subset. However, there is currently no consensus on monocyte subsets and their function, largely because of the lack of standardization of methods to characterize monocytes. Thus, it is important to note that pro-inflammatory and anti-inflammatory properties of monocytes remain difficult to assign to a specific subset [20] and that further research is needed to clarify the role of each subset. In addition, a monoclonal antibody against VLA4 (natalizumab) has been used for over a decade to treat various autoimmune disorders, including multiple sclerosis and Crohn’s disease [33,34]. Natalizumab is a monoclonal antibody that recognizes the α-4 chain (CD49d) of the VLA4 antigen. It acts as an antagonist for VLA4 and prevents leukocyte trafficking into the central nervous system in multiple sclerosis [34]. Given our data on increased VLA4 surface expression on monocytes from APS patients and on monocytes mimicking CAPS, it would be important to further investigate the value of targeting VLA4 antigen in APS. The study of VLA4 would be of utmost importance for the development of treatment alternatives for APS, especially for patients who do not respond to anticoagulation and antiaggregation therapy.

We found no significant difference in surface expression of LFA1, L-selectin MAC1, and PSGL1 on monocytes from APS patients compared with HC. To our knowledge, LFA1, L-selectin, and MAC1 have not been studied in APS, whereas some reports have investigated PSGL1 and the LFA1 counter ligand—ICAM1.

LFA1 is a heterodimer of CD11a and CD18 and binds to its counter ligand ICAM1, inducing monocyte crawling on resting endothelium. No significant differences in plasma levels of ICAM1 were observed in APS patients compared with HC [32], which may be related to the fact that we see no difference in surface expression of LFA1 on monocytes from APS patients. These two adhesion molecules do not seem to be involved in the altered monocyte adhesion during the chronic stage of APS.

PSGL1 binds to all three types of selectins (P-, E-, and L-selectin) on leukocytes. Interaction of selectins with their ligands is normally the first step in the adhesion of monocytes to endothelium under shear stress. The expression of PSGL1 has been previously studied in APS neutrophils. Knight et al. demonstrated overexpression of PSGL1 in neutrophils from APS patients by RNA-seq analysis and showed overexpression of PSGL1 in vitro after stimulation of neutrophils with APS sera [35]. It appears that PSGL1 plays an important role in the binding of APS neutrophils to the endothelium and to platelets, but according to our data, this does not seem to be the case in APS monocytes. Furthermore, in our study, we measured surface expression, which of course, may naturally differ from mRNA expression for a given marker. Interestingly, Bontadi et al. demonstrated significantly higher levels of P-selectin, a PSGL1 receptor, in the plasma of APS patients compared with HC [31]. One would expect that increased levels of P-selectin would also indicate increased levels of its counter ligand (PSGL1) on monocytes, but this was not evident in our data. However, PSGL1 seems to play an important role in APS, as the VNTR polymorphism of PSGL1 appears to be an important determinant of thrombotic predisposition in patients with APS [36]. Thus, further studies are needed to clarify the role of PSGL1 in APS monocytes and their adhesion.

The present study has some limitations. First, it is a preliminary study with a smaller number of patients and samples used. Therefore, larger cohorts are needed to confirm our data, and further approaches to study other adhesion molecules should be performed. The second limitation is the lack of mechanistic support for our results. In the future, we plan to investigate the role of VLA4 in the adhesion of APS and CAPS monocytes to the endothelium also by blocking it and, in addition, to explore the downstream signaling pathways.

## 5. Conclusions

Our preliminary study reports for the first time that VLA4 is a molecule that may play an important role in enhancing adhesion of monocytes to vascular endothelium, thus contributing to the pathogenesis of APS and CAPS. However, further studies are needed to clarify the functional role of VLA4 and its potential as a target, in the treatment of difficult-to-treat APS and CAPS.

## Figures and Tables

**Table 1:** Patients’ demographics, clinical and laboratory features.

	HC (n = 19)	APS (n = 20)
**Demographics**		
Age, median (IQR)	43 (23)	43 (18)
Sex, n (% female)	10 (52%)	11 (55%)
BMI, median (IQR)	25.3 (4.6)	25.9 (4.8)
**Clinical data, n (%)**		
Arterial thrombosis	N/A	6 (30%)
Venous thrombosis	N/A	11 (55%)
Obstetric complications	N/A	3 (15%)
Diabetes	N/A	0 (0%)
Hyperlipidaemia	N/A	9 (45%)
Hypertension	N/A	5 (25%)
**Therapy, n (%)**		
Anticoagulation therapy	N/A	16 (80%)
Antiaggregation therapy	N/A	2 (10%)
Antimalarial medication	N/A	4 (20%)
**Laboratory features, n (%)**		
aCL Ig (G/M/A), n (%)	N/A	14 (70%)
IgG (mean AU ± SD), <10 AU neg	3	19.9 ± 12.0
IgM (AU ± SD), <10 AU neg	3	15.2 ± 10.2
IgA (AU ± SD), <10 AU neg	3	5.9 ± 5.9
anti-β2GPI Ig(G/M/A), n (%)	N/A	13 (65%)
IgG (AU ± SD), <2 AU neg	1	10.4 ± 7.2
IgM (AU ± SD), <2 AU neg	1	2.9 ± 3.0
IgA (AU ± SD), <2 AU neg	1	2.1 ± 1.8
aPS/PT Ig(G/M/A), n (%)	N/A	10 (50%)
IgG (AU ± SD), <5 AU neg	3	39.5 ± 44.3
IgM (AU ± SD), <5 AU neg	3	22.4 ± 31.6
IgA (AU ± SD), <5 AU neg	3	5.1 ± 4.3
Single aPL positive	N/A	4 (20%)
Double aPL positive	N/A	6 (30%)
Triple aPL positive	N/A	10 (50%)
LA positive	N/A	17 (85%)

aCL, anti-cardiolipin antibodies; anti-β2GPI, anti-β2 glycoprotein I antibodies; APS, antiphospholipid syndrome; aPS/PT, anti-phosphatidylserine/prothrombin antibodies; BMI, body mass index; HC, healthy control; IgG, immunoglobulin G; IgM, immunoglobulin M; IgA, immunoglobulin A; IQR, interquartile range; LA, lupus anticoagulant; N/A, not applicable.

**Table 2:** The surface expression of adhesion molecules on circulating peripheral blood monocytes in APS patinets and HCs expressed as percentage of positive cells (IQR).

		Median % Positive (IQR)		
	Molecule	HC (n = 19)	APS (n = 20)	*p*-Value
Total monocytes	LFA1	100 (0.2)	100 (0.0)	0.149
	MAC1	98.9 (2.1)	98.7 (2.2)	0.647
	VLA4	28.8 (16.4)	40.4 (26.6)	0.070
	L-selectin	89.9 (51.7)	85.4 (57.9)	0.967
	PSGL1	99.3 (1.5)	98.6 (1.5)	0.184
Classical subset	LFA1	100 (0)	100 (0)	0.967
	MAC1	99.9 (0.1)	99.9 (0.1)	0.175
	**VLA4**	***23 (14)***	***38.6 (31.2)***	* 0.038
	L-selectin	94 (56.2)	91.6 (61.7)	0.647
	PSGL1	99.9 (0.2)	99.9 (0.4)	0.647
Intermediate subset	LFA1	100 (0.0)	100 (0.0)	1000
	MAC1	*100 (0.3)*	*99.* *8* *(1.* *1* *)*	*0.115*
	**VLA4**	***64.2 (30.4)***	***84.1 (28.4)***	* 0.038
	L-selectin	72.8 (28.9)	62.1 (59.5)	0.184
	PSGL1	100 (0.4)	99.8 (1.1)	0.247
Non-classical subset	LFA1	100 (0.9)	100 (0.1)	0.322
	MAC1	94.2 (18.4)	88.8 (12.8)	0.380
	VLA4	39 (36.6)	36.5 (42)	0.923
	L-selectin	27.2 (25.5)	32.1 (38.3)	0.336
	PSGL1	90.7 (13.7)	87.5 (16.8)	0.258

APS, antiphospholipid syndrome; HC, healthy control; IQR, interquartile range; LFA1, lymphocyte function-associated antigen 1; MAC1, macrophage receptor 1; PSGL1, P-selectin glycoprotein ligand 1; VLA4, very late antigen-4. Statistical differences between the two groups were examined using Mann-Whitney test (* *p* < 0.05).

## Data Availability

Data is contained within the article.
